# Stakeholder engagement to inform HIV clinical trials: a systematic review of the evidence

**DOI:** 10.1002/jia2.25174

**Published:** 2018-10-17

**Authors:** Suzanne Day, Meredith Blumberg, Thi Vu, Yang Zhao, Stuart Rennie, Joseph D. Tucker

**Affiliations:** ^1^ Institute for Global Health and Infectious Diseases University of North Carolina at Chapel Hill Chapel Hill NC USA; ^2^ University of North Carolina – Project China Guangzhou China; ^3^ Department of Social Medicine University of North Carolina at Chapel Hill Chapel Hill NC USA; ^4^ Center for Bioethics University of North Carolina at Chapel Hill Chapel Hill NC USA; ^5^ School of Medicine University of North Carolina at Chapel Hill Chapel Hill NC USA; ^6^ Faculty of Infectious Diseases London School of Hygiene and Tropical Medicine London UK

**Keywords:** stakeholder engagement, community, HIV clinical trials, reporting quality, systematic review, advisory mechanisms

## Abstract

**Introduction:**

Stakeholder engagement is an essential component of HIV clinical trials. We define stakeholder engagement as an input by individuals or groups with an interest in HIV clinical trials to inform the design or conduct of said trials. Despite its value, stakeholder engagement to inform HIV clinical trials has not been rigorously examined. The purpose of our systematic review is to examine stakeholder engagement for HIV clinical trials and compare it to the recommendations of the UNAIDS/AVAC Good Participatory Practice (GPP) guidelines.

**Methods:**

We used the PRISMA checklist and identified English language studies describing stakeholder engagement to inform HIV clinical trials. Four databases (PubMed, Ovid, CINAHL and Web of Science) and six journals were searched, with additional studies identified using handsearching and expert input. Two independent reviewers examined citations, abstracts and full texts. Data were extracted on country, engagement methods, stakeholder types and purpose of stakeholder engagement. Based on the GPP guidelines, we examined how frequently stakeholder engagement was conducted to inform clinical trial research question development, protocol development, recruitment, enrolment, follow‐up, results and dissemination.

**Results and discussion:**

Of the 917 citations identified, 108 studies were included in the analysis. Forty‐eight studies (44.4%) described stakeholder engagement in high‐income countries, thirty (27.8%) in middle‐income countries and nine (8.3%) in low‐income countries. Fourteen methods for stakeholder engagement were identified, including individual (e.g. interviews) and group (e.g. community advisory boards) strategies. Thirty‐five types of stakeholders were engaged, with approximately half of the studies (60; 55.6%) engaging HIV‐affected community stakeholders (e.g. people living with HIV, at‐risk or related populations of interest). We observed greater frequency of stakeholder engagement to inform protocol development (49 studies; 45.4%) and trial recruitment (47 studies; 43.5%). Fewer studies described stakeholder engagement to inform post‐trial processes related to trial results (3; 2.8%) and dissemination (11; 10.2%).

**Conclusions:**

Our findings identify important directions for future stakeholder engagement research and suggestions for policy. Most notably, we found that stakeholder engagement was more frequently conducted to inform early stages of HIV clinical trials compared to later stages. In order to meet recommendations established in the GPP guidelines, greater stakeholder engagement across all clinical trial stages is needed.

## 1 Introduction

Engaging stakeholders in the clinical trial research process has been well established as a method to improve research implementation, procedures, and outcomes 1, 2. Stakeholders can be defined broadly as any individual or group who can have an impact on or is affected by a clinical trial 3. Some examples of stakeholders include trial participants, members of local communities in which a trial is conducted, governmental organizations and funders who shape the research process. Strong stakeholder engagement can potentially result in trials that more effectively address stakeholders’ needs and perspectives 4, as well as improve health equity, access and participant welfare 5. Stakeholder engagement is particularly important in HIV clinical trials, which require careful consideration of the unique physical, psychological and social vulnerabilities associated with HIV infection 6 and subsequent ethical obligations towards trial participants 7. In addition, despite the disproportionate impact of the HIV epidemic on minority communities, these populations are underrepresented in HIV research 8. These factors make stakeholder engagement critical for building effective and sustainable collaborations.

The field of HIV research has championed innovative stakeholder engagement efforts, spurred partly by the activism of those living with HIV. Following the efforts of the ACT‐UP movement, the National Institutes of Health (NIH) established community advisory boards (CABs) in the 1980s to help design and implement research within the NIH trials network, making CABs one of the earliest mandated forms of stakeholder engagement in HIV trials in the United States 9. The first CABs in low‐ and middle‐income countries were established in the late 1990s 10. Since these early efforts, further advancements in stakeholder engagement have included the development of guidance documents such as *Principles of Community Engagement* by the Centers for Disease Control and Prevention 11, as well as guidelines specific to the conduct of HIV research, including: *Respect, Protect, Fulfill*, a guidance document for researchers involving men who have sex with men (MSM) in the HIV research process 12; the *Stakeholder Engagement Toolkit for HIV Prevention Trials* 13; *Recommendations for Community Engagement in HIV/AIDS Research*, developed by community stakeholders partnering with the NIH 14; and the *Good Participatory Practice* (GPP) guidelines for stakeholder engagement in biomedical HIV prevention trials 3. Developed jointly by UNAIDS and the AIDS Vaccine Advocacy Coalition (AVAC) in 2007, the GPP guidelines established a framework for effective stakeholder engagement in HIV clinical trials that are applicable to a broad range of stakeholders and use of an array of engagement methods 3. These guidelines were revised in 2011 based on extensive consultation and feedback with global stakeholders. The GPP guidelines recommend stakeholder engagement as a continual process throughout the stages of a clinical trial: research question development, protocol development, recruitment, enrolment, follow‐up, trial results and dissemination.

Although the importance of stakeholder engagement for HIV clinical trials is widely recognized, little is known about how engagement strategies are being implemented in this field. Existing literature is limited to examining the historical development of stakeholder engagement 15, exploring single sites of stakeholder engagement 16 and reviewing implementation challenges 17. The purpose of our systematic review is to examine stakeholder engagement for HIV clinical trials and compare it to GPP benchmarks. More data on how stakeholder engagement is being conducted in‐practice could help inform GPP guidelines and local engagement strategies for specific HIV trials. Five primary research questions are used to guide our inquiry: (1) What are the geographical locations in which stakeholder engagement is conducted for HIV clinical trials? (2) What methods of stakeholder engagement have been used to inform HIV clinical trials? (3) What types of stakeholders have been engaged? (4) For what purpose has stakeholder engagement been undertaken in relation to informing HIV clinical trials? (5) What is the quality of reporting on stakeholder engagement for HIV clinical trials? By examining how stakeholder engagement for HIV clinical trials has been conducted and reported, our review aims to provide a better understanding of patterns and gaps in existing engagement efforts, pointing to opportunities for improvement in accordance with the recommendations established by the GPP guidelines.

## 2 Methods

### 2.1 Search strategy

**Figure 1:**
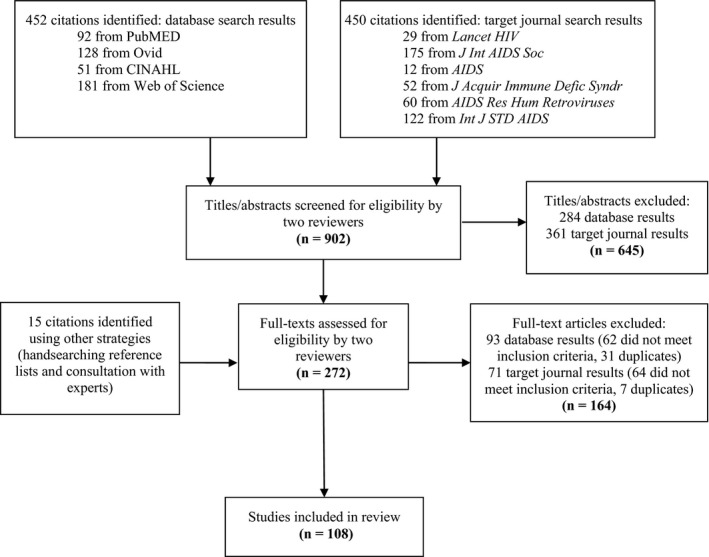
Search and selection strategy results for a systematic review of stakeholder engagement for HIV clinical trials.

We used the PRISMA checklist for reporting systematic review findings (Figure 1). We searched English language studies published before 9 August 2017. Search terms included variations to capture the concept of stakeholder engagement (community engage* OR community consult* OR participatory OR community advis* OR stakeholder*) in combination with the terms HIV and clinical trial*. We searched four databases: PubMed, OVID, CINAHL, and Web of Science. To supplement database results, we additionally searched six HIV journals using their respective journal search functions: *Lancet HIV*,* Journal of the International AIDS Society*,* AIDS*,* Journal of Acquired Immune Deficiency Syndromes*,* AIDS Research and Human Retroviruses* and *International Journal of STD & AIDS*. Finally, studies’ reference lists were handsearched for additional articles to include. We also contacted three individuals with relevant expertise to recommend additional references for inclusion. These individuals were experts in the field of stakeholder engagement for HIV clinical trials and/or principal investigators on NIH‐funded projects examining the conduct of HIV research.

### 2.2 Study selection

To be selected for review, a study had to describe some form of stakeholder engagement undertaken for informing the design or conduct of an HIV clinical trial. Two reviewers independently screened all titles and abstracts returned from searches. Disagreements were resolved through discussion with a third reviewer. The full texts of selected abstracts were then read in full independently by two reviewers for final inclusion and again compared for agreement, with discrepancies resolved by third reviewer. Duplicates were removed and reasons for excluding abstracts and full texts were recorded at each selection stage. A two‐reviewer selection process was similarly applied to studies identified via reference list searching and expert input.

As the purpose of our review was to provide an overview of stakeholder engagement for HIV clinical trials, we used a broad definition of stakeholder engagement. Adapting descriptions of stakeholders and advisory mechanisms for HIV prevention trials outlined in the GPP guidelines 3, we defined stakeholder engagement as any input sought from an individual or group with a stake in HIV clinical trials to inform the design or conduct of said trials. Our definition of clinical trials follows the NIH definition, which encompasses interventions in both biomedical and behavioural outcomes 18. Using this definition allowed us to include studies describing stakeholder engagement to inform both biomedical HIV‐related trials (e.g. vaccine and microbicide trials) and behavioural trials (e.g. trials of behavioural interventions for HIV prevention). Regardless of whether the trial was biomedical or behavioural, it had to be related explicitly to HIV in order to be included in the review; for example, we did not include behavioural trials for prevention of sexually transmitted infections in general.

Recognizing that stakeholder engagement takes place along a continuum from minimal to substantial involvement 2, we did not limit selection of studies based on the extent of stakeholder engagement in the studies identified. We also did not limit inclusion of studies solely to stakeholder engagement efforts undertaken for a current HIV clinical trial; studies describing stakeholder engagement to inform future and/or hypothetical HIV clinical trials (i.e. the field of HIV clinical trial research in general) were also included. Studies were excluded on the basis of not involving stakeholder engagement to inform HIV clinical trials as per our set definitions. We excluded editorials and reviews.

### 2.3 Data extraction and analysis

A data extraction chart was developed to record four characteristics of studies included in the review: the geographical location of engagement activities, the methods used for stakeholder engagement, the types of stakeholders engaged and the purpose of stakeholder engagement. Geographical location was extracted based on the country where stakeholder engagement was conducted using World Bank classifications (high‐, middle‐ or low‐income countries) 19. We did not extract data on the location of the clinical trial given our focus on stakeholder engagement. The purpose of stakeholder engagement refers to the reason that it was undertaken relative to informing the conduct of an HIV clinical trial. Our choice to extract descriptions of purpose rather than outcome was again due to our selection strategy: since we included studies of stakeholder engagement to inform future/hypothetical HIV clinical trials, it was not always possible to extract data on the impact or outcomes of stakeholder engagement. However, all studies described (in varying levels of detail) the purpose for which stakeholder engagement was undertaken relative to inform HIV clinical trials.

Following data extraction, a series of analytic codes were developed to categorize the purpose of stakeholder engagement in each study by the stage of the HIV clinical trial research process that stakeholder engagement was undertaken to inform. Broad coding categories were initially developed based on the seven stages of an HIV clinical trial process as outlined in the GPP guidelines: research question development, protocol development, trial recruitment, participant enrolment, follow‐up, results and dissemination 3. We then used an open coding process to develop and apply thematic codes to each study based on the various reasons for which stakeholder engagement was undertaken to inform any given stage in the HIV clinical trial research process. Coding was conducted exhaustively to categorize the potentially multiple reasons for conducting stakeholder engagement in any one study. For example, if a study included stakeholder engagement to both enhance the ethical conduct of the trial as well as develop effective recruitment strategies, the study would receive both codes.

Data analysis involved comparing our extracted and coded data to three benchmarks outlined in the GPP guidelines 3. First, given that GPP guidelines recommend use of an array of stakeholder advisory mechanisms beyond the clinical trial CABs, we identified and categorized all stakeholder engagement methods used and calculated the number of studies using each method. We did not assess the extent of stakeholder engagement in each study because there are no standardized metrics 20. Second, GPP guidelines stress the identification of relevant trial stakeholders, noting a distinction between community stakeholders (i.e. stakeholders representing the interests of persons participating in the trial and/or affected by the trial) and other stakeholders with interests in HIV clinical trials more broadly (i.e. funders, government representatives). As such, our analysis aimed to identify and categorize the types of stakeholders presently engaged in HIV clinical trials, again calculating the number of studies engaging each stakeholder type. Finally, given that GPP guidelines recommend stakeholder engagement throughout the entire clinical trial, we categorized purpose of engagement according to the seven stages of the HIV clinical trial process.

### 2.4 Quality of reporting on stakeholder engagement

To assess reporting quality, we adapted the short form of a checklist on Guidance for Reporting Involvement of Patients and the Public (GRIPP) in health research 21 into a reporting quality assessment tool. All studies were assessed by one reviewer per study for inclusion of the following information: (1) description of stakeholder engagement purpose; (2) explanation for choice of stakeholder engagement method; (3) description of the development of stakeholder engagement methods used; (4) the number of stakeholders engaged; (5) the results of stakeholder engagement; (6) the impact of stakeholder engagement on trial design/conduct; and (7) discussion of limitations to the stakeholder engagement method used. In each study, these seven reporting details were assessed as being either present or absent. Analysis of reporting quality was conducted for all 108 studies overall, as well as by the type of trial that engagement was conducted to inform (i.e. behavioural prevention trials, biomedical prevention trials, treatment trials or combination/trial type not specified). Additionally, in accordance with the Sex and Gender Equity in Research (SAGER) guidelines 22, we assessed two indicators of the extent to which studies reported on stakeholders’ sex and/or gender (depending on which variable was relevant to the study): the number of stakeholders engaged by sex and/or gender category, and reporting of stakeholder results disaggregated by sex and/or gender.

## 3 Results

As illustrated in Figure 1, a total of 452 titles and abstracts were returned for screening from our four database searches. Of these, 168 full texts were assessed, resulting in 75 studies included for review from the database search strategy. Our target journal search produced 402 titles and abstracts for screening, of which 89 full texts were assessed and 18 studies were retained for review. Our additional search strategies (handsearching of reference lists and inquiry with field experts) yielded an additional 15 studies for inclusion. In total, 108 studies were selected for final inclusion and data extraction. The oldest study included in our review was published in 1988 23, and the most recent was published July 2017 24. Of the 108 studies in our review, 11 studies conducted stakeholder engagement to inform behavioural HIV prevention trials, 70 for biomedical HIV prevention trials and 10 for HIV treatment trials. In the remaining 17 studies, stakeholder engagement was conducted to inform either a combination of HIV‐related trial types or an unspecified type of HIV clinical trial (i.e. HIV clinical trials in general, without specifying whether prevention or treatment, behavioural or biomedical) (see Appendix 1).

Analysis of the data extracted from these 108 studies was guided by our five research questions on the characteristics and reporting of stakeholder engagement to inform HIV clinical trials. The following subsections present in detail the results of each of these five analyses. In terms of the geographical location of stakeholder engagement, the majority of studies in our review described engagement conducted in high‐income countries. Engagement methods used included both individual and group methods. We identified a wide array of stakeholders engaged, ranging from stakeholders directly involved in clinical trial processes to stakeholders on the periphery of HIV clinical trials. Engagement was found to be undertaken much more often for informing earlier stages of HIV clinical trials as compared to later stages. Finally, we found that reporting on the results of stakeholder engagement and limitations associated with engagement are the main gaps in the quality of reporting.

### 3.1 Location of stakeholder engagement

Of the 108 studies, 48 studies (44.4%) conducted stakeholder engagement in high‐income countries 5, 23, 25, 26, 27, 28, 29, 30, 31, 32, 33, 34, 35, 36, 37, 38, 39, 40, 41, 42, 43, 44, 45, 46, 47, 48, 49, 50, 51, 52, 53, 54, 55, 56, 57, 58, 59, 60, 61, 62, 63, 64, 65, 66, 67, 68, 69, 70. In contrast, fewer studies conducted stakeholder engagement in middle‐ (30 studies; 27.8%) 71, 72, 73, 74, 75, 76, 77, 78, 79, 80, 81, 82, 83, 84, 85, 86, 87, 88, 89, 90, 91, 92, 93, 94, 95, 96, 97, 98, 99, 100 and low‐income (nine studies; 8.3%) 101, 102, 103, 104, 105, 106, 107, 108, 109 countries. The location of stakeholder engagement could not be discerned in six studies (5.6%) 110, 111, 112, 113, 114, 115, and fifteen studies (13.9%) 16, 24, 116, 117, 118, 119, 120, 121, 122, 123, 124, 125, 126, 127, 128 conducted stakeholder engagement in multiple countries at different income levels.

### 3.2 Methods of stakeholder engagement for HIV clinical trials

In addition to CABs, we identified 13 other methods of conducting stakeholder engagement across the studies in our review, for a total of 14 distinct methods (Table 1). Methods were separated into five individual methods (i.e. methods involving input or feedback by one stakeholder at a time, such as interviews) and nine group methods (i.e. methods involving input or feedback in a collective format, such as focus groups).

**Table 1:** Methods of stakeholder engagement (n = 108)

Methods of stakeholder engagement		Method description	Studies using each method	Number of studies using each method^a^ (%)	
Individual engagement methods	Stakeholder interviews	Interviews conducted with individuals identified as key stakeholders	5, 16, 26, 27, 28, 32, 33, 44, 49, 54, 56, 58, 62, 68, 69, 72, 76, 79, 82, 86, 88, 89, 90, 91, 93, 95, 96, 97, 98, 100, 101, 102, 106, 108, 109, 110, 111, 113, 116, 117, 124, 125, 128	43 (39.8%)	75 (69.4%)
	Surveys/questionnaires	Surveys or questionnaires about stakeholder perspectives administered by mail, online or in‐person	24, 26, 30, 31, 43, 48, 49, 52, 53, 56, 57, 58, 62, 63, 64, 65, 66, 67, 80, 81, 83, 87, 114, 123	24 (22.2%)	
	Individual stakeholder consultations	Consultations on trial issues/processes sought with specific key informants	30, 34, 40, 45, 50, 72, 103, 104, 105, 118, 119, 120, 121	13 (12.0%)	
	Cognitive mapping	Mixed‐methods approach involving stakeholder interviews, map sketching and observational techniques	77	1 (0.9%)	
	Concept mapping	Mixed‐methods approach involving initial stakeholder idea generation and subsequent stakeholder‐led categorization and ranking of submitted ideas.	35	1 (0.9%)	
Group engagement methods	Focus group discussions	Multiple stakeholders led in a group discussion by a facilitator	5, 16, 27, 32, 33, 37, 38, 39, 45, 46, 47, 50, 55, 60, 71, 74, 79, 82, 85, 89, 90, 94, 96, 98, 100, 101, 106, 107, 108, 109, 116, 125, 128	33 (30.6%)	66 (61.1%)
	Community advisory boards/groups	A formally established group of stakeholders representing community interests and providing a link between trial researchers and the broader community	23, 25, 26, 28, 29, 30, 34, 36, 37, 42, 50, 51, 75, 80, 84, 92, 102, 112, 115, 118, 119, 120, 121, 122	24 (22.2%)	
	Community forums or meetings	Public or invitational meetings held to inform the community about trial issues/processes and obtain feedback from community members	30, 34, 61, 70, 75, 92, 99, 103, 108, 112, 118, 121, 122, 126, 127	15 (13.9%)	
	Stakeholder workshops/education sessions	Events where stakeholders are convened to solve specific trial‐related problem(s) and/or build capacity to understand trial issues/processes	26, 30, 41, 75, 78, 102, 108, 115, 118, 126	10 (9.3%)	
	Community working groups	Group of stakeholders convened to solve or advise on trial‐related problems	59, 75, 120	3 (2.8%)	
	Media outreach campaigns	Informing the broader community about trial issues/processes through mass media and inviting commentary/feedback from stakeholders reached through media messaging	34, 73, 92	3 (2.8%)	
	Crowdsourcing	Having a group participate in solving a problem and then sharing the solution with the public	72	1 (0.9%)	
	Participatory mapping	Community members collaborate with field workers to develop a map depicting local knowledge and community needs	102	1 (0.9%)	
	Dramatic performances	Skits or plays performed to inform about trial‐related issues/processes and prompt feedback from the audience	112	1 (0.9%)	

a For totals and percentages of overall individual versus group methods, studies that used multiple types of individual or group methods were only counted once for each of the two method categories.

As shown in Table 1, individual methods appeared in 75 (69.4%) studies and group methods were used in 66 studies (61.1%). The most frequently used method for stakeholder engagement was stakeholder interviews, followed by focus group discussions. CABs were used as often as surveys/questionnaires. Five methods were used by only one study each: concept mapping 35, cognitive mapping 77, crowdsourcing 72 (having a group participate in solving a problem and then sharing the solution with the public), participatory mapping 102 and dramatic performances 112. All five of these studies were published from the year 2005 onward, suggesting more recent diversification of stakeholder engagement methods. Additionally, many studies used a combination of both individual and group methods for stakeholder engagement; for example, 19 studies (17.6%) paired focus group discussions with stakeholder interviews 5, 16, 27, 32, 33, 79, 82, 89, 90, 96, 98, 100, 101, 106, 108, 109, 116, 125, 128.

### 3.3 Types of stakeholders engaged for HIV clinical trial research

Table 2 presents our analysis of the types of stakeholders engaged throughout all studies reviewed. We identified 35 unique types of stakeholders, which can be grouped into eight subcategories under three broader categories: trial‐related stakeholders, community stakeholders and broader stakeholders. Similar to the categories of stakeholders identified in the GPP guidelines as being relevant to HIV clinical trial research 3, we found that the types of stakeholders engaged ranged from individuals or groups in close proximity to the trial (e.g. trial participants themselves) to broader stakeholders who hold an interest in HIV trial outcomes more generally (e.g. policymakers).

**Table 2:** Types of Stakeholders Engaged (n = 108)

Types of stakeholders engaged			Studies engaging each stakeholder type	Number of studies engaging each stakeholder type^a^ (%)	
Trial‐related stakeholders	Participant trial‐related stakeholders	Trial participants (past or current)	16, 30, 31, 36, 49, 56, 62, 70, 74, 79, 80, 81, 83, 86, 87, 91, 98, 101, 103, 106, 108, 112, 113, 116, 118, 125, 128	27 (25.0%)	29 (26.9%)
		Partners of trial participants	75, 79, 106	3 (2.8%)	
		Potential trial participants (not further specified)	81, 94	2 (1.9%)	
	Non‐participant trial‐related stakeholders	Community advisory board/group members (not further specified)	28, 30, 35, 37, 71, 74, 77, 78, 79, 84, 85, 88, 89, 92, 98, 101, 108, 115, 116, 118, 119, 120, 124, 127	24 (22.2%)	36 (33.3%)
		Trial research team members (e.g. site staff, recruitment officers)	16, 30, 56, 58, 71, 74, 76, 78, 88, 89, 98, 101, 102, 103, 108, 112, 113, 114, 116, 124, 127, 128	22 (20.4%)	
		Trial sponsors	61, 78, 83, 88	4 (3.7%)	
Community stakeholders	HIV‐affected community stakeholders	Populations of interest (e.g. based on race, sexual orientation, occupation, geographical location, risk status)	16, 28, 33, 37, 38, 39, 41, 42, 43, 44, 45, 47, 48, 50, 52, 54, 55, 63, 64, 65, 66, 67, 68, 69, 72, 77, 85, 90, 93, 95, 96, 97, 100, 102, 109, 117, 121, 123, 125, 128	40 (37.0%)	60 (55.6%)
		People involved in HIV advocacy (e.g. community outreach)	16, 23, 25, 28, 30, 33, 34, 36, 51, 57, 61, 78, 96, 100, 105, 118, 127	17 (15.7%)	
		People living with HIV (not further specified)	5, 26, 27, 32, 36, 53, 57, 60, 109	9 (8.3%)	
		Partners of people living with HIV	101	1 (0.9%)	
		Family members/guardians of people living with HIV	23, 104	2 (1.9%)	
	Local community stakeholders	Community leaders (e.g. political, traditional, religious)	5, 16, 23, 29, 32, 36, 45, 60, 75, 82, 93, 96, 98, 100, 102, 105, 106, 109, 112, 121, 122	21 (19.4%)	41 (38.0%)
		Community stakeholders/representatives (not further specified)	29, 30, 56, 59, 70, 92, 98, 108, 112, 120, 122, 125, 128	13 (12.0%)	
		General community members (general public)	26, 30, 46, 48, 72, 73, 82, 92, 99, 107, 115, 121	12 (11.1%)	
		Local media representatives	29, 79, 88	3 (2.8%)	
		School teachers/principals	75, 85	2 (1.9%)	
		Food/recreation facility owners/managers	102	1 (0.9%)	
	Organizational community stakeholders	Non‐governmental organizations	16, 29, 75, 76, 88, 92, 102, 106, 110, 111, 121, 122	12 (11.1%)	20 (18.5%)
		Community‐based organizations/groups serving people living with HIV	5, 16, 26, 32, 36, 79, 82, 111, 118	9 (8.3%)	
		Community‐based organizations (not further specified)	29, 36, 75, 79, 85, 102, 121, 122	8 (7.4%)	
		Human rights groups	111	1 (0.9%)	
Broader stakeholders	Healthcare stakeholders	Healthcare providers	27, 29, 33, 34, 36, 42, 57, 60, 75, 82, 93, 96, 100, 106, 109, 118, 121, 126, 128	19 (17.6%)	23 (21.3%)
		Healthcare facility managers/staff	23, 75, 85, 93, 94, 126	6 (5.6%)	
		Drug industry representatives	61	1 (0.9%)	
	Research stakeholders	IRB/Ethics Committee Members^b^	27, 30, 70, 83, 88	5 (4.6%)	13 (12.0%)
		HIV researchers	24, 61, 76, 118	4 (3.7%)	
		Clinical researchers	70, 94	2 (1.9%)	
		Ethics experts (not further specified)	70, 120	2 (1.9%)	
		Survey design experts	72	1 (0.9%)	
		Research advocates	70	1 (0.9%)	
		Women's health researchers	34	1 (0.9%)	
		Anthropologists	72	1 (0.9%)	
	Governmental stakeholders	Government health research organizations	30, 35, 40, 61, 127	5 (4.6%)	12 (11.1%)
		Government health officials	40, 61, 70, 102, 118	5 (4.6%)	
		Policymakers and government representatives (not further specified)	29, 76, 88, 122	4 (3.7%)	

a For totals and percentages of subcategories of stakeholders, studies that engaged multiple types of stakeholders within the same subcategory were only counted once per subcategory.

b Engagement of IRB/Ethics Committee Members refers to engagement efforts outside of the standard IRB/Ethics review process.

As shown in Table 2, 29 studies included participant trial‐related stakeholders in their engagement efforts and 36 studies engaged non‐participant trial‐related stakeholders, the latter of which included community advisory board/group members, trial staff, and trial funders. Collectively these stakeholders are in closest proximity to the HIV clinical trial research process.

The community stakeholders category presented in Table 2 comprises stakeholders drawn from communities (socially or geographically defined) in which HIV clinical trials may be embedded. Approximately half of all studies included HIV‐affected community stakeholders in their engagement efforts, a subcategory of community stakeholders which encompassed key populations of interest, people involved in HIV advocacy, people living with HIV, and partners and family members of people living with HIV. The most frequently engaged type of stakeholder was populations of interest (40 studies; 37.0%), defined on the basis of socio‐demographic characteristics, occupation, relationship status, HIV risk status or other factors that made a population of particular interest to an HIV clinical trial. In contrast to this targeted approach in defining stakeholders, members of the general public were engaged in just 12 studies (11.1%). Thirteen studies (12%) described engagement conducted with community stakeholders or community representatives without specifying further as to what aspect of the community these stakeholders represent 29, 30, 56, 59, 70, 92, 98, 108, 112, 120, 122, 125, 128.

Stakeholder types encompassed by the broader stakeholders category in Table 2 differ substantially from lay community members. Studies engaging broader stakeholders sought input from medical, academic and governmental experts, including three types of healthcare stakeholders (23 studies), eight types of research stakeholders (13 studies) and three types of governmental stakeholders (12 studies). It is important to note that while these are not the most frequently engaged stakeholders in HIV clinical trials, limiting investigation of engagement to ‘community’ members/representatives would fail to capture the involvement of these groups.

### 3.4 Purpose of stakeholder engagement for HIV clinical trials

Table 3 presents the results of our coding for the purpose of stakeholder engagement in the studies reviewed, organized by the seven stages of HIV clinical trial research.

**Table 3:** Purpose of stakeholder engagement (n = 108)

Purpose of stakeholder engagement, by research stage		Studies using stakeholder engagement for each purpose	Number of studies using each purpose^a^ (%)	
Research question development	Understanding stakeholder perspectives on trial feasibility/acceptability	5, 27, 30, 32, 82, 106, 112, 121, 122	9 (8.3%)	15 (13.9%)
	Setting research priorities/goals	36, 40, 61, 84, 117, 118	6 (5.6%)	
Protocol design	Informing ethical conduct of trial (e.g. participant rights, stopping rules, communication, IRB submission, confidentiality, concepts of fairness)	23, 36, 67, 68, 70, 76, 78, 88, 94, 99, 115, 117, 120, 123, 125, 127, 128	17 (15.7%)	49 (45.4%)
	Developing trial tools (e.g. interventions, measurements, training materials, participant education materials)	24, 25, 29, 35, 36, 37, 38, 39, 42, 72, 74, 103, 106, 107, 119	15 (13.9%)	
	Developing stakeholder engagement strategies for trial	16, 50, 57, 59, 70, 75, 76, 102, 110, 111, 113, 116, 122, 124	14 (13.0%)	
	Developing trial protocol (in general or not further specified)	29, 34, 36, 51, 109	5 (4.6%)	
	Selecting trial sites	34, 37, 105	3 (2.8%)	
	Determining trial participation incentives/compensation	25, 94	2 (1.9%)	
	Securing healthcare services for trial participants	126	1 (0.9%)	
	Developing trial site management strategies	114	1 (0.9%)	
Recruitment	Understanding factors affecting trial recruitment (e.g. attitudes about trial participation)	31, 33, 43, 45, 46, 48, 52, 53, 54, 55, 58, 60, 62, 63, 64, 65, 66, 69, 85, 87, 89, 90, 95, 97, 98, 100, 106, 109, 110	29 (26.9%)	47 (43.5%)
	Building community education/awareness to enhance recruitment and/or community support for trial	26, 29, 30, 41, 73, 75, 92, 112, 115, 121, 122	11 (10.2%)	
	Developing trial recruitment strategies	29, 34, 47, 50, 68, 77, 93	7 (6.5%)	
	Building credibility for trial among community to enhance recruitment	37	1 (0.9%)	
Enrolment	Enhancing the informed consent process	49, 53, 56, 71, 80, 81, 96, 99, 101, 108, 112, 120, 128	13 (12.0%)	13 (12.0%)
Follow‐up	Developing retention strategies	34, 36, 50, 77, 93, 104	6 (5.6%)	17 (15.7%)
	Understanding factors affecting trial adherence/retention	28, 31, 44, 79, 91	5 (4.6%)	
	Addressing participants’ concerns as they arise in trial	75, 108, 118, 122	4 (3.7%)	
	Understanding participants’ expectations about the trial	86	1 (0.9%)	
	Building community education/awareness to enhance retention	115	1 (0.9%)	
Results	Developing post‐trial processes (e.g. post‐trial access to medication)	62, 83	2 (1.9%)	3 (2.8%)
	Reviewing/interpreting trial results	29	1 (0.9%)	
Dissemination	Communicating results to broader stakeholders	23, 25, 36, 110, 117, 118, 122	7 (6.5%)	11 (10.2%)
	Communicating results to trial participants	62, 75, 86, 110, 118, 122	6 (5.6%)	
	Developing academic products based on trial results	29	1 (0.9%)	

a For totals and percentages by research stage, studies that conducted stakeholder engagement for multiple purposes within the same research stage were only counted once per research stage.

We identified 25 distinct purposes for which stakeholder engagement was undertaken. The most frequently reported purpose for stakeholder engagement was for understanding factors affecting trial recruitment (29 studies). This includes studies that examined how stakeholders’ attitudes about HIV trial participation may impact recruitment; for example, examining how stakeholders’ perceptions of early trial termination might affect willingness to participate in future vaccine trials 33, 62. Additional examples of studies using stakeholder engagement for this purpose include studies investigating barriers and facilitators to trial participation among specific populations 55, 87. The second and third most frequent purpose for conducting stakeholder engagement was to inform the ethical conduct of the trial (16 studies) and to develop trial tools (15 studies) respectively. In informing the ethical conduct of trials, stakeholders were engaged for providing input on ethics‐related concerns, either in terms of the overall trial process 70, 76, 88, 99 or in relation to particular aspects of the trial; for example, trial stopping rules 78, trial communication strategies 123 and concepts of fairness in the research relationship 94.

**Figure 2:**
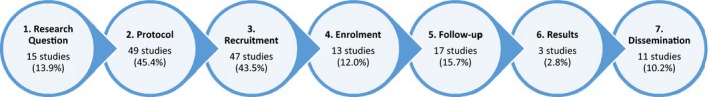
Summary of the purpose of stakeholder engagement by clinical trial research stage.

By examining the purpose of stakeholder engagement by research stage in Table 3, we observed that stakeholder engagement was conducted more often to inform the earlier stages of trials. More studies described undertaking stakeholder engagement to inform the trial protocol development stage (49 studies; 45.4%) than any other research stage. Nearly the same volume of studies (47; 43.5%) undertook stakeholder engagement to inform trial recruitment. Stakeholder engagement to inform the final two stages of the research process was described least frequently, with just three studies engaging stakeholders to inform the trial results stage and eleven to inform dissemination of trial results. This disparity in studies conducting stakeholder engagement for purposes across the seven stages of research is more clearly visualized by Figure 2.

### 3.5 Quality of stakeholder engagement reporting

Table 4 summarizes the results of our assessment of reporting quality, indicating the number of studies meeting seven criteria adapted from the GRIPP2 checklist to improve reporting of stakeholder involvement in health research 21. We also disaggregated our analysis of stakeholder engagement reporting quality by the type of HIV‐related trial that the stakeholder engagement was meant to inform (see Appendix 1).

**Table 4:** Quality of stakeholder engagement reporting (n = 108 studies)

Reporting quality criteria		Studies meeting reporting quality criteria	Number of studies (%)
Aim	Describes the purpose of stakeholder engagement	5, 16, 23, 24, 25, 26, 27, 28, 29, 30, 31, 32, 33, 34, 35, 36, 37, 38, 39, 40, 41, 42, 43, 44, 45, 46, 47, 48, 49, 50, 51, 52, 53, 54, 55, 56, 57, 58, 59, 60, 61, 62, 63, 64, 65, 66, 67, 68, 69, 70, 71, 72, 73, 74, 75, 76, 77, 78, 79, 80, 81, 82, 83, 84, 85, 86, 87, 88, 89, 90, 91, 92, 93, 94, 95, 96, 97, 98, 99, 100, 101, 102, 103, 104, 105, 106, 107, 108, 109, 110, 111, 112, 113, 114, 115, 116, 117, 118, 119, 120, 121, 122, 123, 124, 125, 126, 127, 128	108 (100%)
Methods	Explains reasons for choice of stakeholder engagement method(s)	23, 27, 29, 35, 36, 38, 39, 41, 63, 69, 72, 73, 77, 78, 81, 86, 88, 92, 99, 101, 102, 104, 105, 106, 115, 116, 120, 121, 122, 124	30 (27.8%)
	Describes development of engagement method(s) used	5, 16, 23, 26, 27, 29, 30, 32, 33, 34, 35, 36, 38, 39, 40, 41, 43, 44, 45, 46, 47, 48, 49, 52, 53, 54, 55, 56, 57, 58, 59, 60, 62, 63, 64, 66, 69, 70, 72, 73, 75, 77, 78, 79, 80, 81, 82, 83, 85, 86, 87, 88, 89, 90, 92, 93, 94, 95, 96, 97, 98, 99, 100, 101, 102, 103, 104, 105, 106, 108, 109, 110, 111, 114, 116, 119, 120, 122, 124, 125, 126, 128	82 (75.9%)
	Reports the number of all stakeholders engaged	16, 24, 25, 26, 27, 28, 29, 30, 31, 32, 33, 35, 36, 38, 39, 40, 43, 44, 45, 46, 47, 48, 49, 52, 53, 54, 55, 56, 57, 59, 60, 62, 63, 64, 65, 66, 67, 68, 69, 71, 74, 75, 76, 77, 78, 79, 80, 81, 82, 83, 85, 86, 87, 88, 89, 90, 91, 92, 93, 94, 95, 96, 97, 98, 99, 100, 101, 102, 103, 106, 108, 109, 110, 111, 113, 114, 116, 123, 124, 125, 127, 128	82 (75.9%)
Results	Describes results of stakeholder engagement	5, 16, 23, 24, 26, 27, 28, 29, 30, 31, 32, 33, 34, 35, 36, 38, 39, 40, 41, 43, 44, 45, 46, 48, 49, 52, 53, 54, 55, 56, 57, 58, 59, 60, 61, 62, 63, 64, 65, 66, 67, 68, 69, 70, 71, 72, 73, 74, 75, 76, 77, 78, 79, 80, 81, 82, 83, 85, 86, 87, 88, 89, 90, 91, 92, 93, 94, 95, 96, 97, 98, 99, 100, 101, 102, 104, 105, 106, 108, 109, 110, 111, 113, 114, 116, 117, 118, 119, 120, 121, 122, 123, 124, 125, 126, 127, 128	97 (89.8%)
Outcomes^a^	Discusses impact of stakeholder engagement on HIV clinical trial (where applicable)	23, 25, 29, 30, 31, 34, 36, 39, 41, 47, 49, 50, 61, 72, 73, 75, 78, 92, 103, 104, 105, 108, 109, 115, 118, 119, 120, 121, 122	29 (70%)^a^
Reflections	Discusses limitations of stakeholder engagement	5, 16, 24, 26, 27, 28, 29, 31, 32, 33, 34, 35, 36, 41, 43, 44, 45, 46, 48, 52, 53, 54, 55, 56, 58, 60, 62, 63, 64, 68, 71, 73, 74, 75, 76, 78, 79, 80, 81, 82, 83, 87, 88, 90, 91, 93, 94, 95, 96, 98, 99, 100, 101, 102, 110, 111, 114, 116, 120, 123, 125, 127	62 (57.4%)

a Reporting on outcomes was assessed only among 41 studies that were not related to future/hypothetical trials.

While all 108 studies included in the review described at least one purpose for conducting stakeholder engagement, Table 4 demonstrates that most studies also provided details on the development of the engagement methods, the number of stakeholders engaged and the results of stakeholder engagement. ‘Results of stakeholder engagement’ refers to reporting the information obtained through a study's engagement method, such as reporting findings from focus group discussions 27, 71, 101. This differs from reporting the *outcome* of stakeholder engagement, which we defined as reporting on the impact that stakeholder engagement made on the design or conduct of an HIV clinical trial, such as describing how the results of crowdsourcing were subsequently used to develop a clinical trial's intervention 72. We found that stakeholder engagement outcomes were assessable among 41 studies (67 studies included in the review conducted stakeholder engagement to inform future/hypothetical trials); however, among these 41 studies, only 29 (70%) met the reporting criteria, meaning 30% of studies with the opportunity to report on stakeholder engagement outcomes did not do so. Additionally, of all 108 studies reviewed, 60 (55.6%) reported the number of engaged stakeholders by sex and/or gender category; however, only four studies reported stakeholder engagement results disaggregated by sex and/or gender category 38, 52, 74, 87.

## 4 Discussion

This systematic review described stakeholder engagement for HIV clinical trials and compared this engagement to GPP recommendations. Our review suggests critical gaps in stakeholder engagement that should be examined and addressed in the field of HIV clinical trial research.

First, we found more of the studies included in our review conducted stakeholder engagement in HICs compared to LMICs. This finding is consistent with a review of clinical trial priority setting processes 129. One potential explanation for these results may be that there is a greater proportion of HIV clinical trials conducted in HIC settings, as a review of infectious disease trials registered with ClinicalTrials.gov found that the greatest proportion of all registered HIV trials were located in North America and Europe 130. In addition, conducting stakeholder engagement in LMICs may be hindered by limited resources, communication barriers, and mistrust of research 16. However, while stakeholder engagement may be challenging to conduct in LMICs, these are also the contexts in which stakeholder engagement may be most important 131. Our results demonstrate a need for more evidence to inform HIV clinical trials in LMICs.

Second, our data suggest that while many methods are used, most stakeholder engagement is conducted using researcher‐driven, top‐down methods. This often involves formal social science methods such as in‐depth interviews or focus group discussions. It is unclear how effective these methods are for fostering meaningful partnerships and continuous dialogue as the GPP guidelines recommend 3. Additionally, the extent to which top‐down engagement methods can inform the design and conduct of HIV clinical trials depends entirely on trial researchers. Thus, while the GPP guidelines recommend that trial researchers carefully consider and select from the range of possible advisory mechanisms 3, the reliance on top‐down, expert‐driven stakeholder engagement suggests the need for these and other guidance documents to consider innovative, bottom‐up engagement strategies. For example, crowdsourcing approaches that allow community members a more participatory role in informing HIV clinical trials could supplement existing stakeholder engagement strategies 132. Engagement methods that follow a participatory model can help to achieve more meaningful inclusion of stakeholders and greater opportunities to change the status quo 2.

Third, we found that stakeholder engagement was predominately conducted to inform early trial stages. These findings are comparable to those of studies examining stakeholder engagement in other fields of health research 133, 134. Both of these studies emphasize the importance of engaging stakeholders throughout all stages of the research, a recommendation also posed by the GPP guidelines for HIV clinical trials 3. In order to meet these benchmarks for GPP, our results suggest that greater efforts are particularly needed to engage stakeholders in the later stages of HIV clinical trial research. Future research should examine innovative methods to foster opportunities for stakeholder contributions at these points in the research process. Additionally, while multiple guidance documents exist to promote meaningful and effective stakeholder engagement 3, 11, 12, 13, 14, HIV clinical trial teams should consider how to tailor these recommendations so that engagement efforts account for the specificities of the type of trial being conducted as well as for local contexts (e.g. social, political). These efforts by HIV researchers could help to establish models for stakeholder engagement in clinical trial research more broadly.

Our findings should be considered alongside broader factors that inform the engagement process and researcher‐stakeholder relationship in HIV clinical research. The extent to which stakeholders are engaged is shaped not only by the clinical trial team, but also by the structural contexts within which clinical trial research is embedded. As noted by others 15, it is important to consider how funders and corporate interests influence stakeholder engagement. For example, the funding of many HIV trials by high‐income countries may inadvertently assert norms and activities (e.g. community advisory boards) that are not locally driven. The impact of global resource disparities on stakeholder engagement should also be considered, particularly for the potential to reproduce inequalities in terms of which stakeholders are engaged 16. Thus, while the results of our review help to make visible some of the gaps in current stakeholder engagement for HIV clinical trials, more research is needed to account for *why* these gaps occur and how best to address these gaps as a product of broader structural contexts.

There are several important limitations to this review. First, we did not assess quality of engagement. However, there is a notable lack of quality measurement tools for stakeholder engagement 20, as well as disagreement regarding whether and how to determine what level of engagement is appropriate 135. Second, our review does not examine the outcomes of stakeholder engagement; however, only 41 studies (38%) in our review provided information on engagement outcomes. Future reviews should focus on systematically assessing engagement outcomes in relation to methods used and stakeholders engaged. Third, our finding that fewer studies conducted stakeholder engagement in LMICs may be attributable in part to our search strategy being limited to English language studies only. Manuscript selection bias (i.e. the overrepresentation of scientific publications from HICs) may also play a role 136. Fourth, the extent to which this review can provide an overview of stakeholder engagement for HIV clinical trials is necessarily dependent on the extent to which these activities are reported. It is possible that more engagement takes place “behind the scenes” of clinical trial research without making its way into published accounts of trial results. Improved reporting standards in accordance with guidance documents such as those used in our analysis of reporting quality 21 may help to provide further evidence for all research teams seeking to enhance their own engagement approaches, regardless of HIV trial type.

## 5 Conclusions

The results of this systematic review of stakeholder engagement for HIV clinical trials have implications for research and policy. First, our finding of fewer studies conducting stakeholder engagement in LMICs suggests the need for further reporting on stakeholder engagement in these settings 131. Additional resources and regulations to support and sustain stakeholder engagement in these settings may be necessary to address potential barriers to engagement. Second, despite engagement recommendations outlined in comprehensive guidelines 3 and funding allocated on the part of national and international funding bodies to support engagement activities 20, our findings suggest that stakeholder engagement is not being conducted evenly to inform all stages of the HIV clinical trial process. More research is needed in order to understand barriers and facilitators to involving stakeholders in the later stages of HIV clinical trial research specifically, as well as which methods of engagement would be most conducive to involving stakeholders in trial results and dissemination processes. Funders should additionally consider adding specifications to stakeholder engagement requirements to help address this gap, such as requiring clinical trial researchers to include detailed engagement plans for each stage of the trial process. Future research could then examine whether and how stakeholder engagement changes over time in response to such efforts. Finally, to address gaps identified in reporting quality, HIV research journals should consider implementing policies about reporting stakeholder engagement. Checklists for reporting on stakeholder engagement 21, 133 may help to promote greater transparency as to what engagement efforts are undertaken in trials and how this engagement shapes the research process. This information will be particularly valuable for undertaking future research to evaluate the quality of stakeholder engagement.

## Competing interests

The authors declare that they have no competing interests.

## Authors’ contributions

S.D., J.T. and R.S. conceived of the idea for this review. S.D. designed the review protocol, and S.D. and M.B conducted the search. S.D., Y.Z., M.B. and T.V read and selected the citations, abstracts and full texts. S.D., Y.Z. and T.V extracted the data. S.D. with assistance from M.B. and T.V., as well as in consultation with S.R. and J.T conducted the coding and analysis. S.D., M.B. and T.V., with substantial contributions and edits provided by Y.Z., S.R. and J.T, prepared the manuscript. All authors reviewed, provided feedback and approved the final manuscript.

## Authors’ information

The authors are part of a working group examining the social and ethical aspects of research on curing HIV (searcHIV). More information about our working group is available at: http://searchiv.web.unc.edu/

## Supporting information


**Appendix S1.** Quality of Reporting on Stakeholder Engagement, by Type of HIV Clinical Trial.
