# Supplementary material for: Stakeholder engagement to inform HIV clinical trials: a systematic review of the evidence
Source: J Int AIDS Soc. 2018 Oct 17;21(Suppl Suppl 7):e25174. doi: 10.1002/jia2.25174 (PMC6192899; doi:10.1002/jia2.25174)
Supplement: Supplementary file 1 — Appendix S1. Quality of Reporting on Stakeholder Engagement, by Type of HIV Clinical Trial. [file JIA2-21-e25174-s001.docx]

**Appendix 1: Quality of Reporting on Stakeholder Engagement, by Type of HIV Clinical Trial**

| **Reporting Quality Criteria** | | **Studies Meeting Reporting**  **Quality Criteria** | | | | | | | |
| --- | --- | --- | --- | --- | --- | --- | --- | --- | --- |
|  |  | Behavioral Prevention Trials | Number of Studies (%) | Biomedical Prevention Trials | Number of Studies (%) | Treatment Trials | Number of Studies (%) | Combination/Not Specified | Number of Studies (%) |
| **Aim** | Describes the purpose of  stakeholder engagement | [24, 27, 35-38, 47, 58, 72, 105, 120] | 11 (100%) | [16, 23, 26, 29, 30, 32, 43-46, 48, 49, 51, 52, 54-56, 59, 62-71, 73-76, 78-82, 84-93, 95-98, 100, 102, 103, 106, 108-111, 115, 117-119, 121-123, 125-128] | 70 (100%) | [25, 33, 39, 42, 53, 60, 83, 94, 104, 107] | 10 (100%) | [5, 28, 31, 34, 40, 41, 50, 57, 61, 77, 99, 101, 112-114, 116, 124] | 17 (100%) |
| **Methods** | Explains reasons for choice of stakeholder engagement  method(s) | [35, 37, 38, 72, 105, 120] | 6 (54.5%) | [26, 63, 69, 73, 78, 81, 86, 88, 92, 102, 106, 115, 121, 122] | 14 (20%) | [39, 104] | 2 (20%) | [28, 34, 41, 77, 99, 101, 116, 124] | 8 (47.1%) |
|  | Describes development of engagement method(s) used | [35, 37, 38, 47, 58, 72, 105, 120] | 8 (72.7%) | [16, 26, 29, 32, 43-46, 48, 49, 52, 54-56, 59, 62-64, 66, 69, 70, 73, 75, 78-82, 85-90, 92, 93, 95-98, 100, 102, 103, 106, 108-111, 119, 122, 125, 126, 128] | 53 (75.7%) | [25, 33, 39, 53, 60, 83, 94, 104] | 8 (80%) | [5, 28, 31, 34, 40, 41, 57, 77, 99, 101, 114, 116, 124] | 13 (76.5%) |
|  | Reports the number of all stakeholders engaged | [24, 27, 35, 37, 38, 47] | 6 (54.5%) | [16, 23, 26, 29, 30, 32, 43-46, 48, 49, 52, 54-56, 59, 62-69, 71, 74-76, 78-82, 85-93, 95-98, 100, 102, 103, 106, 108-111, 123, 125, 127, 128] | 59 (84.3%) | [25, 53, 60, 83, 94] | 5 (50%) | [28, 31, 34, 40, 57, 77, 99, 101, 113, 114, 116, 124] | 12 (70.6%) |
| **Results** | Describes results of stakeholder engagement | [27, 35, 37, 38, 58, 72, 105, 120] | 8 (72.7%) | [16, 23, 26, 29, 30, 32, 43-46, 48, 49, 52, 54-56, 59, 62-71, 73-76, 78-82, 85-93, 95-98, 100, 102, 106, 108-111, 117-119, 121-123, 125-128] | 66 (94.3%) | [25, 33, 39, 53, 60, 83, 94, 104] | 8 (80%) | [5, 28, 31, 34, 40, 41, 57, 61, 77, 99, 101, 113, 114, 116, 124] | 15 (88.2%) |
| **Outcomes*** | Discusses impact of stakeholder engagement on HIV clinical trial (where applicable) | [24, 35, 38, 47, 72, 105, 120] | 7  (9 assessable; 77.8%) | [29, 30, 49, 73, 75, 78, 92, 103, 108, 109, 115, 118, 119, 121, 122] | 15  (23 assessable; 65.2%) | [33, 39, 104] | 3  (5 assessable; 60%) | [28, 41, 50, 61] | 4  (4 assessable; 100%) |
| **Reflections** | Discusses limitations of stakeholder engagement | [27, 35, 58, 120] | 4 (36.4%) | [16, 23, 26, 30, 32, 43-46, 48, 52, 54-56, 62-64, 68, 71, 73-76, 78-82, 87, 88, 90, 91, 93, 95, 96, 98, 100, 102, 110, 111, 123, 125, 127] | 43 (61.4%) | [25, 33, 53, 60, 83, 94] | 6 (60%) | [5, 28, 31, 34, 41, 99, 101, 114, 116] | 9 (52.9%) |

* Reporting on outcomes was assessed only among studies that were not related to future/hypothetical trials.
